# A Rare Case of Severe Post-operative Pyoderma Gangrenosum After Surgery for Perforated Diverticulitis at the Sigmoid Colon

**DOI:** 10.7759/cureus.35807

**Published:** 2023-03-06

**Authors:** Yoko Shono, Akinori Sekioka, Shuichi Ota, Tetsuo Ito, Yukito Adachi

**Affiliations:** 1 Gastroenterological Surgery, Osaka Saiseikai-Noe Hospital, Osaka, JPN; 2 Gastroenterological Surgery, Osaka Saiseikai-Noe hospital, Osaka, JPN

**Keywords:** pathergy, idiopathic, tumor necrosis factor-α(tnf-α) inhibitor, post-operative, pyoderma gangrenosum

## Abstract

Pyoderma gangrenosum (PG) is a nonbacterial ulcerating skin condition. It is typically associated with other systemic disorders. However, approximately 20%-30% of cases are idiopathic. Post-operative PG (PPG) is a rare type of PG with a rapidly expanding cutaneous ulcer at a surgical site and is often misdiagnosed as a wound infection. The difficulty in diagnosis can lead to unnecessary surgical interventions and delay in the treatment of PG.

Herein, we present the case of a 68-year-old patient with severe PPG with no underlying diseases. He underwent an emergency laparotomy (Hartmann’s procedure) for perforated diverticulitis. After the operation, systemic inflammatory response syndrome (SIRS) developed and the skin around the incisional wound, stoma, injection venous lines, and electrocardiogram monitoring pads gradually became erythematous. Skin biopsy and the absence of a source of infection confirmed the diagnosis of PG. Drug therapy for PG with steroids, and tumor necrosis factor-α inhibitors improved SIRS and the patient recovered.

## Introduction

Pyoderma gangrenosum (PG) is a chronic, idiopathic, rapidly evolving cutaneous ulcerative condition. It is a non-infectious dermatosis characterized by painful erythematous papules, pustules, or vesicles that rapidly develop into ulcers with ragged, undermined, violaceous, or gunmetal-colored borders [1-3]. Approximately 50% of PG are associated with various autoimmune diseases, such as rheumatoid arthritis or inflammatory bowel disease (IBD), whereas almost 30% cases are idiopathic [1,4]. Post-operative PG (PPG) is a subtype of PG with ulcerative dermatosis, which is often confused with wound infection following surgery [5]. This difficulty in diagnosis can result in unreasonable debridement and delays in appropriate treatment [6]. Here, we present a rare case of severe PPG developing after an abdominal surgery that was efficiently treated by early diagnosis and appropriate medication.

## Case presentation

A 68-year-old man (168 cm, 68 kg, BMI 24.1 kg/m^2^) experienced abdominal pain and presented to a local clinic. The patient had a history of hypertension, but no history of autoimmune disorders and skin symptoms following other trauma or injuries in life. Since bacterial colitis was suspected, the patient received oral antibiotic treatment. Subsequently, during the following days, the symptoms and blood examination results worsened, and he was referred to our hospital.

**Figure 1:**
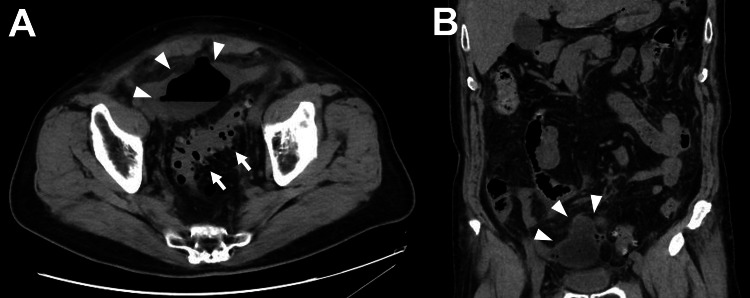
Abdominal computed tomography (A) Axial computed tomography (CT) showing several diverticula at the sigmoid colon (arrow), with inflammation, surrounded by free air, and ascites (arrowheads). (B) Coronal CT showing severe inflammation in the lower abdominal abscess (arrowheads)

On general examination, his blood pressure was 140/82 mmHg, heart rate 83 bpm, body temperature 37.4 °C (99.3 °F), and peripheral oxygen saturation on room air 97%. Abdominal examination suggested pan-peritoneal inflammation, with tapping pain and Blumberg’s sign. Laboratory test results demonstrated elevation of inflammatory markers: white blood cell (WBC) count 8,600/μL (normal value 3,500-8,000/μL), C-reactive protein (CRP) 37.3 mg/dL (normal value < 0.3 mg/dL), procalcitonin (PCT) 1.0 ng/mL (normal value < 0.046 ng/mL). We used CRP as the degree of inflammation in this case. Computed tomography (CT) showed several diverticula at the sigmoid colon, with inflammation, surrounded by free air and ascites (Figures 1A, 1B).

**Figure 2:**
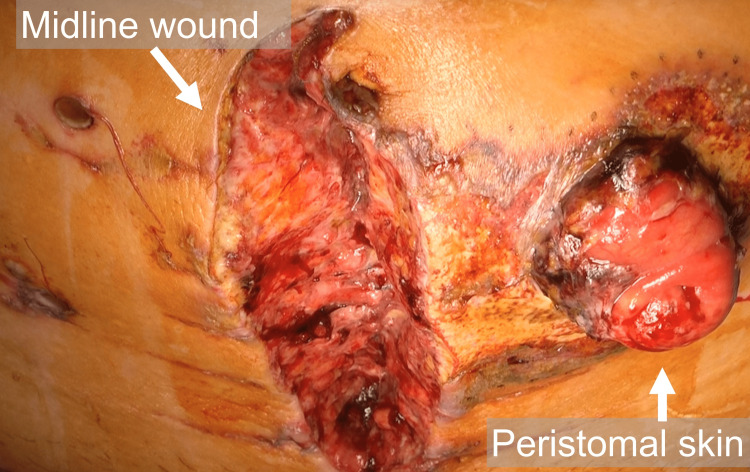
Midline abdominal wound and peristomal skin became erythematous, dehiscent, and ulcerated

He was diagnosed with severe diverticulitis resulting in colonic perforation and generalized peritonitis; and underwent emergency surgery with Hartmann’s procedure and abscess drainage. Post-operatively, the patient developed systemic inflammatory response syndrome (SIRS) and disseminated intravascular coagulation (DIC), probably due to severe inflammation in the abdominal cavity, which was empirically treated by broad-spectrum antibiotics (meropenem, teicoplanin, and micafungin). Endotracheal intubation was performed, and he was put on an artificial ventilator. On post-operative day (POD) 4, blood tests revealed worsening of the inflammation (WBC 24,100/μL, CRP 45.2 mg/dL, PCT 1.5ng/mL), and the surgical wound, including the peristomal skin became erythematous and ulcerated and dehiscent (Figure 2).

Since CT on POD 5 revealed ascites in the abdominal cavity, which suggested a post-operative abscess causing severe inflammation, we performed an emergency laparotomy for drainage. However, only mild contamination of the abdominal cavity was revealed, without any source of infection. The initial stoma was removed, and another loop stoma was made in the transverse colon.

**Figure 3:**
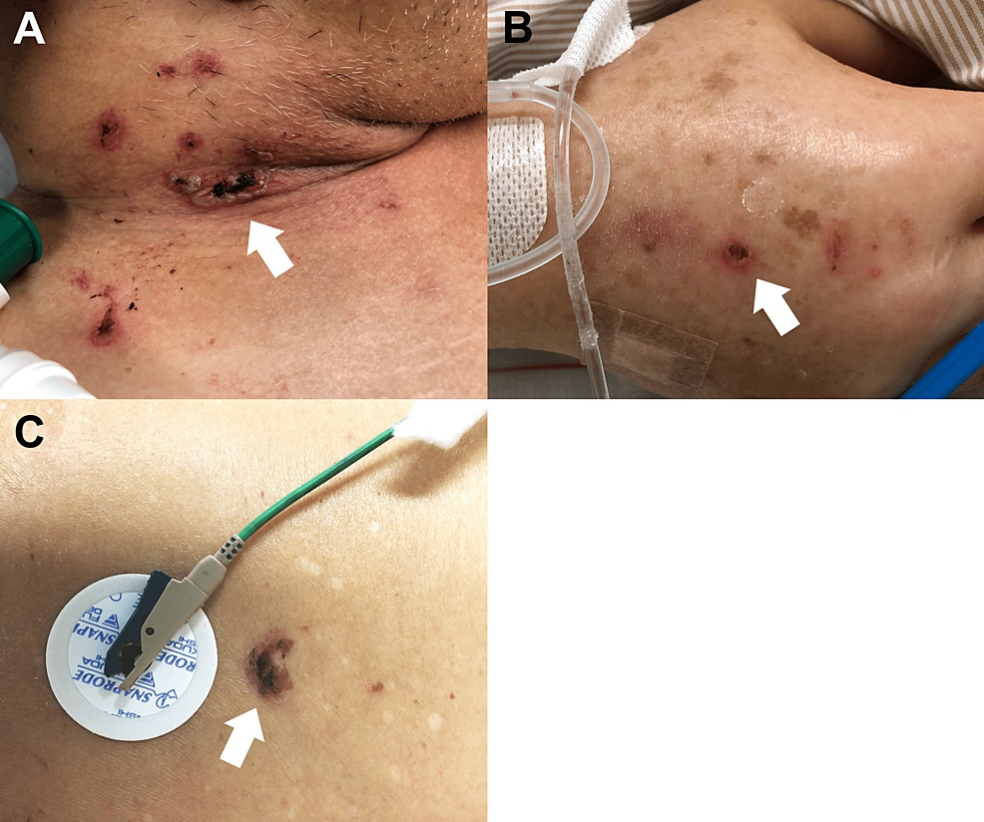
The sites of pathergy (A) The scar of the central venous catheter in the right internal jugular vein (arrow), (B) the scar of the venous line (arrow), and (C) the scar of electrocardiogram monitor pads (arrow) became erythematous.

SIRS was not relieved after the second operation. At this time, along with the lesion around the incisional scar the skin adjacent to the attached electrocardiogram pads and intravenous injection sites became erythematous (Figures 3A-3C).

Those characteristics suggest the pathergy phenomenon. Additionally, all bacterial culture tests from blood (obtained on POD 0, 2), wound (on POD 5, 6), sputum (on POD 5), and ascites (obtained during the second surgery) were negative. Pathological findings of resected colon revealed only diverticulitis and no evidence of IBD. At this stage, PPG was suspected as the cause of SIRS. Dermatologists performed a skin biopsy around the erythematous wound, and histological examination showed neither vasculitis nor neoplastic cells. It showed neutrophilic invasion in the dermis. Considering these findings, we diagnosed PG.

**Figure 4:**
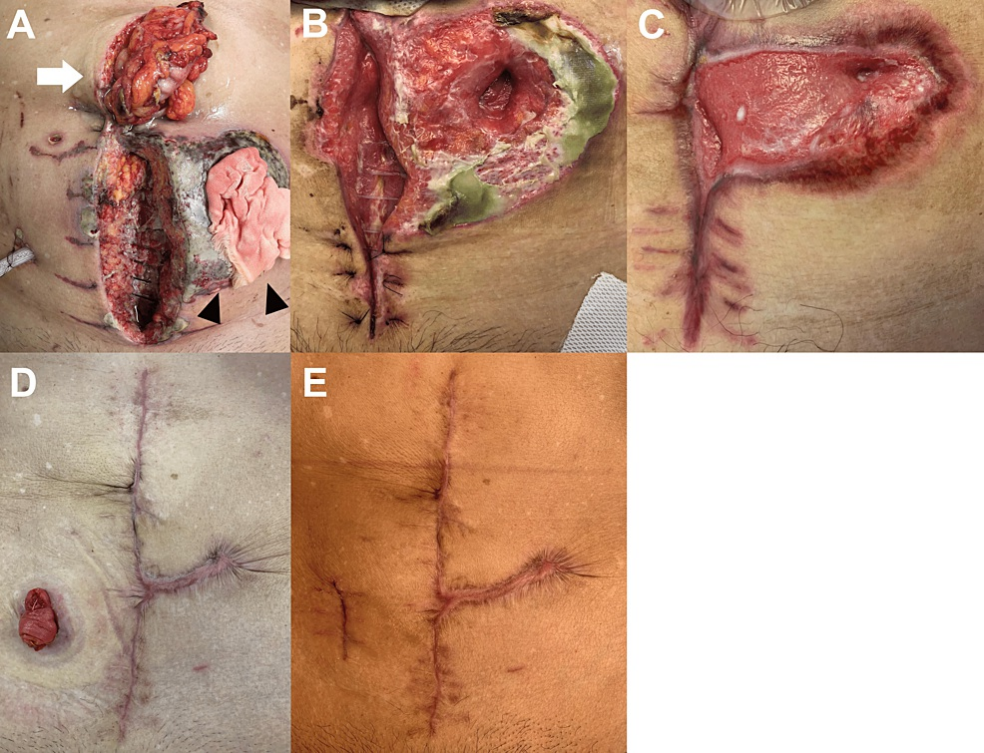
The wound condition (A) The wound condition after the second operation. A transverse colon loop stoma was created, and the initial sigmoid colon stoma was removed. Because the skin around the wound was inflamed and necrotic, it remained open. (B) The wound condition on POD 36. (C) The wound condition on POD 101, at the time of discharge. (D) Seven months after the first operation, the patient underwent colonic anastomosis with temporary ileostomy in good wound condition. (E) The wound condition after the ileostomy was closed one year after the first operation.

As the treatment for PG, intravenous prednisolone 70 mg (1 mg/kg) every 24 hours was initiated. In the following days, the patient’s general condition and SIRS gradually improved, and he was extubated on POD 18 and left the ICU on POD 20. The combination of intravenous prednisolone and tumor necrosis factor-α (TNF-α) inhibitor; adalimumab, was introduced on POD 28. After the introduction of adalimumab, the dose of prednisolone could be tapered by 5 mg per week. He was discharged on POD 101 as the skin condition had improved (Figures 4A-4C).

After discharge, the patient’s skin condition and inflammation stabilized with oral prednisolone (gradually reduced to 4 mg/day) and a weekly subcutaneous injection of adalimumab. Seven months after the first operation, the patient underwent colonic anastomosis with temporary ileostomy under steroid cover (Figure 4D). We used prednisolone (4 mg/day) as perioperative steroid cover, and adalimumab was withdrawn for two weeks before and after surgery. Three months later, the ileostomy was closed (Figure 4E). Two years after the initial operation, the patient was in good condition without regression of PG symptoms and was free of prednisolone and adalimumab.

## Discussion

PG is a rare inflammatory skin disorder characterized by cutaneous papules or pustules that evolve rapidly into large, painful, necrotic, and non-bacterial ulcers with violaceous, undermined edges [5]. Association with the systemic disease has been reported in 50% of cases; most commonly with IBD, rheumatoid arthritis, and hematological disorders, while approximately 30% are idiopathic [7]. Furthermore, the activity of the underlying disease may reflect the severity of PG [8,9].

The diagnosis of PG has been challenging because it depends mainly on clinical symptoms and the exclusion of other diseases [10]. A recent report suggested useful diagnostic criteria for PG, in which there are one major and eight minor criteria [10]. Based on these, our case included one major criterion: biopsy of the ulcer edge demonstrating neutrophilic infiltrate, and six minor criteria: (1) exclusion of infection; (2) pathergy phenomenon; (3) history of papules, pustules, or vesicles ulcerating within 4 days of appearance; (4) peripheral erythema, undermined border, and tenderness at ulceration sites; (5) “wrinkled paper” scars at healed ulcer sites; and (6) decrease in ulcer size within 1 month of initiating immunosuppressive medications. Pathergy is defined as the development of skin lesions at sites of injury. The frequency of pathergy in PG is 20%-30%. It is considered useful for suspecting PG [11].

PPG refers to the development of PG at surgical sites within a short time post-operatively [12]. A systematic review reported that 8.6% of patients who developed PPG had a hematologic disorder, 5.9% of patients had IBD, and 3.6% had rheumatoid arthritis [5], while approximately 50% of the PPG are completely idiopathic [13]. When clinicians encounter a case of PPG, they commonly consider the ulcer or erythematous skin to be a wound infection. Therefore, it can lead to unnecessary surgical intervention such as debridement, which would exacerbate symptoms and delay the treatment for PG further, and the condition of the patient could become more severe and, in the worst-case scenario, life-threatening.

The initial therapy for mild PG is the local application of steroids, while systemic treatment is the most common intervention for severe PG [14,15]. Systemic steroid administration has been the first-line therapy for moderate or severe PG because of the rapid response it has [16]. As second-line or adjunctive therapies, immunomodulators, TNF-α inhibitors, mycophenolate mofetil, azathioprine, or methotrexate are used in combination with steroids. In recent reports, the benefits of TNF-α inhibitors, infliximab, and adalimumab, have been mentioned [17-19].

In our case, systemic treatment was introduced from the beginning owing to the severity of PG and SIRS. Specifically, we administered prednisolone at 1 mg/kg/day and reduced the dose at a rate of 5 mg per five days because of the development of steroid psychosis. Adalimumab (160 mg) was subsequently administered as the first dose 21 days after prednisolone initiation. Two weeks after the first subcutaneous injection of adalimumab, we administered adalimumab at 80 mg. Two weeks later, we administered adalimumab at 40 mg once a week. No complications related to adalimumab were observed during the clinical course.

Although there are a few previous reports of PG with SIRS, it rarely causes a life-threatening situation [20]. It is suggested that a delay in the diagnosis of PG could result in the patient facing a critical situation. To the best of our knowledge, the present case is the first report of idiopathic PPG with SIRS that was completely resolved. Despite the critical inflammation, early diagnosis and treatment of PG enabled us to save the patient.

## Conclusions

This is a rare case report of PPG with SIRS after abdominal surgery. The exclusion of infectious sources, atypical dermatosis, and subsequent skin biopsy confirmed the diagnosis of PPG. We believe that a relatively early diagnosis of PG and treatment with a combination of steroids and TNF-α inhibitors could be used to treat this life-threatening condition.

## References

[1] Jiang YY, Li J, Li Y (2017). Comparison of clinical features between pyoderma gangrenosum concomitant by inflammatory bowel disease and idiopathic pyoderma gangrenosum. Chin Med J (Engl).

[2] Tagliaferri AR (2020). Post-operative pyoderma gangrenosum: a long journey for a patient with myelodysplastic syndrome. Cureus.

[3] Tolkachjov SN, Fahy AS, Wetter DA (2015). Postoperative pyoderma gangrenosum (PG): the mayo clinic experience of 20 years from 1994 through 2014. J Am Acad Dermatol.

[4] Saracino A, Kelly R, Liew D, Chong A (2011). Pyoderma gangrenosum requiring inpatient management: a report of 26 cases with follow up. Australas J Dermatol.

[5] Zuo KJ, Fung E, Tredget EE, Lin AN (2015). A systematic review of post-surgical pyoderma gangrenosum: identification of risk factors and proposed management strategy. J Plast Reconstr Aesthet Surg.

[6] Chen ZQ, Huang JF, Ma LL, Zhang CL, Lei S (2018). Usefulness of metagenomic analysis in differential diagnosis for pyoderma gangrenosum. J Int Med Res.

[7] Powell FC, Schroeter AL, Su WP, Perry HO (1985). Pyoderma gangrenosum: a review of 86 patients. Q J Med.

[8] Sheldon DG, Sawchuk LL, Kozarek RA, Thirlby RC (2000). Twenty cases of peristomal pyoderma gangrenosum: diagnostic implications and management. Arch Surg.

[9] Hasselmann DO, Bens G, Tilgen W, Reichrath J (2007). Pyoderma gangrenosum: clinical presentation and outcome in 18 cases and review of the literature. J Dtsch Dermatol Ges.

[10] Maverakis E, Ma C, Shinkai K (2018). Diagnostic criteria of ulcerative Pyoderma Gangrenosum a delphi consensus of international experts. JAMA Dermatol.

[11] Callen JP, Jackson JM (2007). Pyoderma gangrenosum: an update. Rheum Dis Clin North Am.

[12] Tolkachjov SN, Fahy AS, Cerci FB, Wetter DA, Cha SS, Camilleri MJ (2016). Postoperative pyoderma gangrenosum: a clinical review of published cases. Mayo Clin Proc.

[13] Almukhtar R, Armenta AM, Martin J, Goodwin BP, Vincent B, Lee B, Dacso MM (2018). Delayed diagnosis of post-surgical pyoderma gangrenosum: a multicenter case series and review of literature. Int J Surg Case Rep.

[14] Reichrath J, Bens G, Bonowitz A, Tilgen W (2005). Treatment recommendations for pyoderma gangrenosum: an evidence-based review of the literature based on more than 350 patients. J Am Acad Dermatol.

[15] Le Cleach L, Moguelet P, Perrin P, Chosidow O (2011). Is topical monotherapy effective for localized pyoderma gangrenosum?. Arch Dermatol.

[16] Miller J, Yentzer BA, Clark A, Jorizzo JL, Feldman SR (2010). Pyoderma gangrenosum: a review and update on new therapies. J Am Acad Dermatol.

[17] Brooklyn TN, Dunnill MG, Shetty A (2006). Infliximab for the treatment of pyoderma gangrenosum: a randomised, double blind, placebo controlled trial. Gut.

[18] Yamamoto T (2021). An update on adalimumab for pyoderma gangrenosum. Drugs Today (Barc).

[19] Nakayama Y, Akeda T, Iida S (2021). Whether to maintain or strengthen the treatment for pyoderma gangrenosum ulcerative type may depend on the response after two to four-week treatment intervention: the outcome of three cases with details clinical course. Clin Case Rep.

[20] Osaka A, Ide H, Ban S (2018). Pyoderma gangrenosum after radical prostatectomy: case report. Int Cancer Conf J.

